# Relationship between health-related quality of life and respiratory health status among coal-based sponge iron plant workers in Barjora, India

**DOI:** 10.1007/s10389-017-0861-9

**Published:** 2017-11-06

**Authors:** Mousumi Biswas, Kaushik Chattopadhyay

**Affiliations:** 1 Centre for Reviews and Dissemination, University of York, York, YO10 4DU UK; 2 Division of Epidemiology and Public Health, School of Medicine, The University of Nottingham, Nottingham, UK

**Keywords:** Health-related quality of life, Respiratory health status, EQ5D, SGRQ

## Abstract

**Background:**

Many coal-based sponge iron plant workers have poor health-related quality of life in general, and specifically a poor respiratory health status. However, the relationship between their health-related quality of life and respiratory health status is unknown.

**Aim:**

This study investigated the relationship between health-related quality of life, measured using the EuroQol-5D (EQ5D), and respiratory health status, measured using the St. George’s Respiratory Questionnaire (SGRQ), among coal-based sponge iron plant workers in Barjora, India.

**Method:**

A cross-sectional study was conducted among coal-based sponge iron plant workers in Barjora, and complete data were available on 252 participants. Spearman’s rank correlation coefficients were reported to show the strength of relationship between health-related quality of life and respiratory health status.

**Results and conclusion:**

Significant correlations were found between all EQ5D dimensions/visual analogue scale (VAS) and all SGRQ scores except between EQ5D-VAS and SGRQ-activity. A range of correlations was found. They were moderate between EQ5D-anxiety/depression and SGRQ-symptom, EQ5D-VAS and SGRQ-symptom, and EQ5D-anxiety/depression and SGRQ-total, but weak between all the other factors.

## Background

During the past decade, the number of coal-based sponge iron plants has grown rapidly in the Barjora block of Bankura district, a deprived district in West Bengal, India [Centre for Science and Environment (CSE) 2011]. These factories are categorised as red industries (i.e. highly polluted industries) with the major pollutants including particulate matters and gaseous pollutants, the toxic effects of which are often rapid (Centre for Science and Environment (CSE) 2011; Cerana Foundation 2006; Chatterjee 2011; Patra et al. 2012). Prolonged exposure to such pollutants leads to obstructive lung disease such as asthma or chronic obstructive respiratory disease (COPD) (Gizaw et al. 2016; Lai and Christiani 2013). Coal-based sponge iron plant workers are not only at risk of developing respiratory diseases, but many also have some form of respiratory disease (Chattopadhyay 2016). The unhealthy working environment is evidently a significant predictor of health-related quality of life (Taghavi et al. 2014). Coal-based sponge iron plant workers have poor health-related quality of life and respiratory health status (Chattopadhyay et al. 2014; Chattopadhyay et al. 2015). Poor health-related quality of life and respiratory health status place a burden on the individual, family, community and health services, and are thus of major public health importance (Fayers and Machin 2007; Institute of Medicine 2001; Jones 1995; World Health Organization (WHO) 2001).

A single intervention can only be developed, evaluated and implemented to improve the health-related quality of life of people as well as their respiratory health status if they are found to be related. Previous studies have shown a significant association between psychological factors (such as anxiety and depression) and respiratory health status in people with respiratory diseases (Balcells et al. 2010; Dyer et al. 1999). Psychological components are an important part of the health-related quality of life (Fayers and Machin 2007). Some research has been conducted to explore the correlation between health-related quality of life and the respiratory health status of patients with respiratory diseases such as COPD or idiopathic pulmonary fibrosis (IPF) (Chen et al. 2014; Starkie et al. 2011; Wilke et al. 2012) and found significant relationships. Studies have also been conducted in different countries to explore the health-related quality of life and respiratory health status in various industrial workers, but the relationship between their health-related quality of life and respiratory health status has never been explored (Rachiotis et al. 2006; Zhu et al. 2012). Similarly, the relationship between the health-related quality of life and respiratory health status among coal-based sponge iron factory workers is unknown. Hence, the aim of this study was to investigate the relationship between health-related quality of life [measured using the EuroQol-5D (EQ5D)] and respiratory health status [measured using the St. George’s Respiratory Questionnaire (SGRQ)] among coal-based sponge iron plant workers in Barjora.

## Methods

### Study

This article presents a new analysis of a study conducted among coal-based sponge iron plant workers in Barjora (Bankura district, West Bengal, India) in May and June 2013 to assess their health-related quality of life and respiratory health status (Chattopadhyay et al. 2014; Chattopadhyay et al. 2015). We analysed the complete data available on 252 participants to report the relationship between health-related quality of life and respiratory health status. Ethics approval was received from the Barjora Block Development Office Committee and was based on the Indian Council of Medical Research Ethical Guidelines for Biomedical Research on Human Participants, 2006 [Indian Council of Medical Research (ICMR) 2006].

### Description of EQ5D

Participants’ health-related quality of life was assessed using the EQ5D questionnaire (Rabin et al. ; The EuroQol Group. 1990). The EQ5D assesses the overall functioning and well-being of individuals in terms of their mobility, self-care, usual activities, pain or discomfort, and anxiety or depression. In addition, participants rate their current health using a visual analogue scale (VAS) on a 0 (death or worst possible health) to 100 (best possible health) scale.

### Description of SGRQ

Participants’ respiratory health status was assessed using the St. George’s Respiratory Questionnaire (SGRQ). The SGRQ has 50 items with 76 weighted responses (Jones et al. 1991) and provides three domain scores (symptoms, activity and impact) and a total score, ranging from 0 (optimal) to 100 points (worst).

### Statistical analysis

The five dimensions of the EQ5D were described in terms of percentages. The EQ5D-VAS, all three SGRQ domains and SGRQ total scores were described as means and standard deviations (SD). Only participants who completed all aspects of these two questionnaires were included in the analyses. Spearman’s rank correlation coefficients (*r*_*s*_) were reported to show the relationship between EQ5D and SGRQ. The strength of the relationship was defined as strong (*r*_*s*_ > 0.701), moderate (*r*_*s*_ = 0.301 to 0.700) or weak (*r*_*s*_ < 0.300). Data analyses were done using Stata 14 for Windows software (StataCorp 2015).

## Results

All 252 participants were males, with a mean age of 35.4 years (SD 8.2). The majority of the participants were manual workers (78%) and literate (88%).

**Fig. 1:**
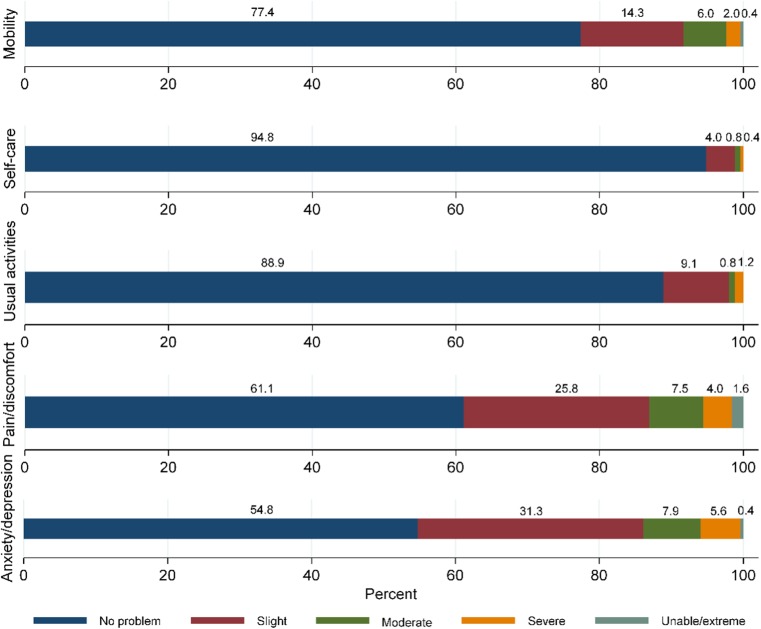
Five dimensions of EQ5D - percentages

Percentages for the five EQ5D dimensions are shown in Fig. 1. In the mobility dimension, 77% of participants responded that they had no problem with mobility, and 23% responded that they had some problem (14% slight, 6% moderate and 2% severe with 0.4% unable to walk). In the self-care dimension, most of the participants had no problem taking self-care (95%) and only a few participants (5%) had some problems taking self-care. In the usual activities dimension, the majority of the participants responded that they had no problem performing usual activities (89%) and only 11% responded that they had some problems performing usual activities (9% slight, 1% moderate and 1% severe). In the pain/discomfort dimension, 61% of participants reported that they had some form of pain or discomfort (26% slight, 8% moderate, 4% severe and 2% extreme). In the anxiety/depression dimension, almost half of the participants reported slight to extreme anxiety or depression (31% slight, 8% moderate, 6% severe and 0.4% extreme).

For the EQ5D-VAS, the three SGRQ domain scores and the SGRQ total score, means and SDs are presented in Table 1. The mean EQ5D-VAS score was 69.9 (SD 18.6). The mean scores in the SGRQ domains were 9.9 for SGRQ-symptom (SD 18.4), 9.6 for SGRQ-activity (SD 19.0) and 5.9 for SGRQ-impact (SD 13.8). The mean total SGRQ score was 7.7 (SD 14.6).

**Table 1:** EQ5D-VAS and SGRQ scores - means and SDs

Domains	Mean (SD)
EQ5D-VAS	69.9 (18.6)
SGRQ-symptoms	9.9 (18.4)
SGRQ-activity	9.6 (19.0)
SGRQ-impact	5.9 (13.8)
SGRQ-total	7.7 (14.6)

*SD* Standard deviation

Spearman’s rank correlation coefficients (*r*_*s*_) are presented in Table 2 to show the relationship between EQ5D and SGRQ. Correlations between all EQ5D dimensions/VAS and all SGRQ scores were significant except between EQ5D-VAS and SGRQ-activity. A negative correlation was observed between the EQ5D-VAS and all SGRQ scores with *r*_*s*_ ranging between −0.196 and −0.349. The strength of these correlations varied. It was moderate between EQ5D-anxiety/depression and SGRQ-symptom (*r*_*s*_ = 0.440), EQ5D-VAS and SGRQ-symptom (*r*_*s*_ = −0.349), and EQ5D-anxiety/depression and SGRQ-total (*r*_*s*_ = 0.341). All other factors had a weak relationship with *r*_*s*_ ranging between 0.133 and 0.291.

**Table 2:** Relationship between EQ5D dimensions and EQ5D-VAS and SGRQ domains

	SGRQ-symptoms *r*_*s*_ (*p-value*)	SGRQ-activity *r*_*s*_ (*p-value*)	SGRQ-impact *r*_*s*_ (*p-value*)	SGRQ-total *r*_*s*_ (*p-value*)
Mobility	0.135 (0.032)	0.140 (0.026)	0.158 (0.012)	0.148 (0.012)
Self-care	0.229 (<0.001)	0.136 (0.031)	0.247 (<0.001)	0.249 (<0.001)
Usual activities	0.214 (0.001)	0.178 (0.005)	0.233 (<0.001)	0.227 (<0.001)
Pain/discomfort	0.268 (<0.001)	0.133 (0.034)	0.195 (0.002)	0.237 (<0.001)
Anxiety/depression	0.440 (<0.001)	0.173 (0.006)	0.291 (<0.001)	0.341 (<0.001)
EQ5D-VAS	−0.349 (<0.001)	−0.071 (0.262)	−0.196 (0.002)	−0.236 (<0.001)

*r*_*s*_= Spearman’s rank correlation coefficient

## Discussion

Significant correlations were found between all EQ5D dimensions/VAS and all SGRQ scores except between EQ5D-VAS and SGRQ-activity. A range of correlations was found: moderate between EQ5D-anxiety/depression and SGRQ-symptom, EQ5D-VAS and SGRQ-symptom, and EQ5D-anxiety/depression and SGRQ-total, but weak between all the other factors. A statistically significant negative relationship was observed between EQ5D-VAS and the SGRQ domains (except SGRQ-activity). Perhaps this observed negative relationship was the result of how these two measurement tools were scored. A higher value in the EQ5D-VAS reflected a better health-related quality of life, whereas a lower value in the SGRQ reflected a better respiratory health status (Jones et al. 1991; The EuroQol Group. 1990).

Our study showed a weak correlation between EQ5D-VAS and the SGRQ-total score (Spearman’s rank correlation coefficient −0.236, *p*-value < 0.001). It included participants who were at risk of developing respiratory diseases as well as those who already had some form of a respiratory disease (Chattopadhyay 2016). However, a previous study conducted among COPD patients in Hong Kong showed a moderate correlation between EQ5D-VAS and the SGRQ-total score (Pearson correlation coefficient −0.437, p-value < 0.001) (Chen et al. 2014). In this study, more than half of the participants had severe COPD and participants, on average, had been diagnosed with COPD for 7 years. The advanced nature and severity of the disease and associated prolonged suffering could have influenced this moderate correlation. Another study investigated the correlation between the EQ5D index score and the SGRQ-total score in patients with advanced COPD in the Netherlands (Wilke et al. 2012). This study showed a strong correlation between the EQ5D index score and the SGRQ-total score at baseline and moderate correlation at 4, 8 and 12 months. However, the study did not report any correlation between the EQ5D dimensions/VAS and SGRQ domains. Therefore, the study results are not comparable with our study.

Ours is the first study to investigate the correlation between the health-related quality of life and respiratory health status of coal-based sponge iron plant workers. Our study proves that respiratory health status could be a potential predictor of health-related quality of life among coal-based sponge iron plant workers and vice versa. As they are related, a single intervention could be developed, evaluated and implemented to improve their health-related quality of life as well as their respiratory health status.

This study has some limitations that could restrict the generalisability of our results. First, the study was conducted among coal-based sponge iron factory workers in a particular location and it remains unknown whether similar significant relationships would be observed among coal-based sponge iron factory workers in other parts of India or worldwide. Second, it was not possible to determine the relationship between health-related quality of life and respiratory health status over time, as this was a cross-sectional study. A longitudinal study is needed to evaluate such a relationship over time. Finally, all the participants were male; therefore, any gender-related differences in health-related quality of life and respiratory health status might not be reflected. Despite these limitations, this study contributes to the ongoing research on the health-related quality of life and respiratory health status among coal-based sponge iron factory workers.

## Conclusion

In conclusion, significant correlations were found between all EQ5D dimensions and EQ5D-VAS and all SGRQ scores, except between EQ5D-VAS and SGRQ-activity. A range of correlations was found: moderate between EQ5D-anxiety/depression and SGRQ-symptom, EQ5D-VAS and SGRQ-symptom, and EQ5D-anxiety/depression and SGRQ-total, but weak between all the other factors.
